# l-Lactate Dehydrogenase B Chain Associated with Milk Protein Content in Dairy Cows

**DOI:** 10.3390/ani9070442

**Published:** 2019-07-15

**Authors:** Tao Wang, Seung Woo Jeon, U Suk Jung, Min Jeong Kim, Hong Gu Lee

**Affiliations:** 1 Department of Animal Science and Technology, College of Animal Bioscience and Technology, Konkuk University, Seoul 143-701, Korea; 2 Department of Animal Nutrition and Feed Science, College of Animal Science and Technology, Jilin Agricultural University, Changchun 130118, China

**Keywords:** milk epithelial cells, l-leucine, MAC-T cells, milk protein content, l-lactate dehydrogenase B chain

## Abstract

**Simple Summary:**

The aim of this study was to explore the genes associated with milk protein content in dairy cows. Seven down-regulated and three up-regulated proteins were found in isolated milk epithelial cells (MECs) from dairy cows with high milk protein level (group High). l-leucine depletion not only decreased the proteins synthesis (*p* < 0.05), but also decreased l-lactate dehydrogenase B chain (LDH-B) mRNA expression in bovine mammary alveolar cells MAC-T cells (*p* < 0.05). This study suggested that LDH-B is negatively associated with the milk protein content of dairy cows and has a positive relationship with l-leucine. These findings may provide some theoretical basis to study individual differences in the milk protein synthesis ability of dairy cows.

**Abstract:**

This study aimed to explore genes associated with milk protein content in dairy cows and their relationships with l-leucine. Ten primiparous Holstein cows (93.8 ± 11.56 milking days) fed the same diet were divided into two groups depending on their milk protein contents (group High, 3.34 ± 0.10%; and group Low, 2.86 ± 0.05%). Milk epithelial cells (MECs) were isolated from the collected morning milk and differentially expressed proteins in MECs were explored by two-dimensional gel electrophoresis (2-DE). Then, the mRNA expression of these proteins was detected by real time PCR in MAC-T cells incubated with three different media named positive control (PC), negative control (NC), and l-leucine depletion (NO-leu). Results showed that ten proteins were differentially expressed in MECs from cows in group High. They included seven down-regulated ones (heat shock protein beta-1 (HSPB1), 78 kDa glucose-regulated protein (GRP-78), l-lactate dehydrogenase B chain (LDH-B), malate dehydrogenase, cytoplasmic (MDH1), annexin I (ANXA1), cytokeratin-7 (CK-7), and glyceraldehyde-3-phosphate dehydrogenase (GAPDH)), and three up-regulated ones (prohibitin (PHB), beta casein (CSN2), and alpha S1 casein (CSN1S1)). When l-leucine was depleted from the medium, not only proteins content was lowered (*p* < 0.05), but also the LDH-B mRNA expression was decreased in MAC-T cells (*p* < 0.05). In conclusion, LDH-B is negatively associated with the milk protein content of dairy cows and has a positive association with l-leucine.

## 1. Introduction

In milk, about 25% of total milk solids are proteins that are of great importance to milk processors for the manufacturing of a range of products [1]. Therefore, milk protein seems to be a more valuable component than others in milk. However, milk protein contents are diverse in traditional cow breeds of economic importance. For instance, the milk protein level of Holsteins was lowest (3.15% to 3.25%), while the Jersey tends to be the highest (3.80% to 3.90%) [2,3]. Not only are there significant differences in milk protein profiles and frequencies of casein genotype in different breeds [4], the individual contents of milk proteins in the same breed are various too [3,5]. Studies have indicated that fatty acid composition in milk can be changed by feeding, while protein composition is not significantly influenced by the cow’s diet [3,5]. Some amino acids were found not only serving as substrate, but also acting as signaling molecules for protein synthesis [5,6]. l-leucine, one of the branched-chain amino acids, is recognized as a major regulator in protein synthesis [7,8]. However, the mechanism involved in leucine-specific signaling is still not well elucidated and needs further study. We hypothesized that some proteins related to l-leucine are associated with the milk protein content of dairy cows. Therefore, in order to improve the understanding of leucine-specific signaling in milk protein synthesis, proteins differentially expressed were explored in isolated bovine milk epithelial cells (MECs) from cows with high milk protein level, and their relationships with l-leucine were checked in bovine mammary alveolar cells (MAC-T cells) in this study.

## 2. Materials and Methods

### 2.1. Milk Preparation and Milk Epithelial Cells (MECs) Isolation

Ten primiparous Holstein cows (573 ± 24.6 kg body weight; 93.8 ± 11.56 milking days) fed with the same total mixed ration (TMR) were selected from a commercial dairy farm. The cows were milked twice daily (12 h milking interval) and 1.5 L milk was individually collected from the automatic milking machine in the morning. Milk samples were divided into two groups (group High, 3.34 ± 0.10%; group Low, 2.86 ± 0.05%) according to milk protein contents analyzed by MilkoScan (Foss Electric, Hillerød, Denmark) [9]. Then, milk epithelial cells (MECs) were separated from the samples using anti-cytokeratin 8 antibodies (Sigma-Aldrich, St. Louis, MO, USA) attached to magnetic beads (Pan Mouse IgG; Invitrogen Dynal AS, Oslo, Norway) within 24 h [10]. All of the experimental animals were approved by Animal Welfare and Ethics Committee of Jilin Agricultural University and managed according to the Guidelines for the Care and Use of Experimental Animals.

### 2.2. Analysis of Protein Expression

Concentrations of proteins extracted from isolated MECs of these two groups (group High and group Low) were measured with a commercial Kit (GE Healthcare, Piscataway, NJ, USA). Global protein expression was compared using a two-dimensional gel electrophoresis (2-DE) system. The isoelectric focusing (IEF) was performed by using an IEF electrophoresis unit (GE Healthcare, Piscataway, NJ, USA), while the SDS-PAGE was performed by using an Ettan DALT 2-D gel system (GE Healthcare, Piscataway, NJ, USA). After electrophoresis, the gels were silver-stained using a PlusOne Silver Staining kit (GE Healthcare, Piscataway, NJ, USA) and processed by Proteomweaver™ 2-D Analysis software (Definiens AG, Munich, Germany). Differently expressed proteins (≥2-fold or ≤0.5-fold) were picked for ESI-Q-TOF/MS analysis and proteins identifications [10].

### 2.3. l-Leucine Depletion Experiment in MAC-T Cells

MAC-T cells were seeded into 6-well cell culture plates (BD Falcon, Becton, Dickinson and Company, Mississauga, ON, Canada) and cultured with complete DMEM/F12 media (pH = 7.4) (Thermo Scientific, South Logan, UT, USA) at 2 mL/well. When 80% confluency was reached, the media was replaced with serum-free complete DMEM/F12 (Gibco, Invitrogen, Grand Island, NY, USA) and incubated overnight. After washing twice with PBS, cells were incubated with one of the following three media: (1) Earle’s Balanced Salts Solution (Gibco, Invitrogen, Grand Island, NY, USA) + insulin (5 μg/mL) + hydrocortisone (1 μg/mL) + prolactin (5 μg/mL) (Sigma-Aldrich Corp, St. Louis, MO, USA) as negative control (NC); (2) negative control medium supplemented with l-arginine (0.70 mmol/L), l-histidine (0.15 mmol/L), l-isoleucine (0.42 mmol/L), l-leucine (0.45 mmol/L), l-lysine (0.5 mmol/L), l-methionine (0.12 mmol/L), l-phenylalanine (0.22 mmol/L), l-threonine (0.45 mmol/L), l-tryptophan (0.04 mmol/L), and l-valine (0.45 mmol/L) as positive control (PC) [7]; and (3) PC media devoid of l-leucine as l-leucine depletion (NO-leu) group. The NO-leu medium was prepared from NC medium by adding nine individual AAs (l-isomer) at concentrations equal to that of PC medium. After incubation for 1 or 6 h, media and cells were collected individually. Total proteins were calculated as the sum of proteins in cell and proteins secreted into the culture media.

### 2.4. The mRNA Expression of Candidate Proteins in MAC-T Cells

Total RNA was extracted from MAC-T cells using TRIZOL reagent according to the manufacturer’s instructions (Thermo Fisher Scientific, Waltham, MA, USA), and the RNA quality was assessed by Agilent 2100 Bioanalyzer (Agilent Technologies Inc, Santa Clara, CA, USA). The real-time PCR was performed with a total reaction volume of 20 µL in 96-well plates using the CFX96 Real-Time PCR Detection System (Bio-Rad Laboratories, Hercules, CA, USA). β-actin was used as housekeeping gene. The primer sequences are shown in Table 1. Detailed procedure of real-time PCR was reported by Wang et al. (2014) [11].

### 2.5. Statistical Analysis

All data were presented as mean ± standard error (S.E.). The data of milk protein contents of dairy cows were analyzed with *t*-test while the data of total proteins content and mRNA expression in MAC-T Cells were analyzed with one-way analysis of variance (one-way ANOVA, SPSS Inc., Chicago, IL, USA). Differences were considered significant when *p* < 0.05.

## 3. Results

### 3.1. Milk Protein Contents for Individual Animals

**Figure 1:**
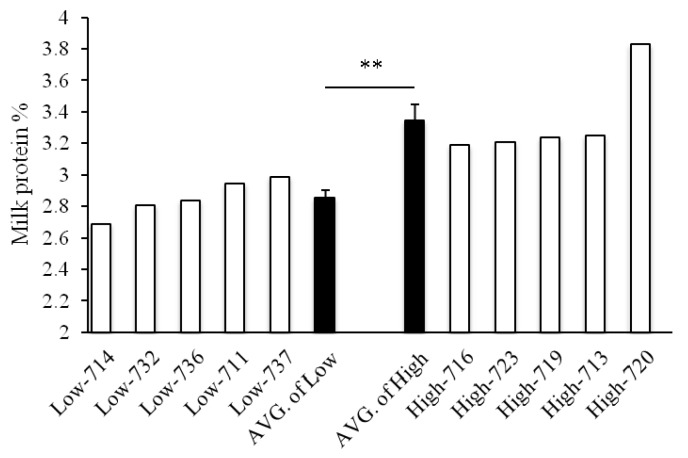
Milk protein contents of primiparous dairy cows (93.8 ± 11.56 milking days). ** *p* < 0.01.

In this study, milk protein contents from individual primiparous Holstein cows in the same lactation stage were quite different, ranging from a minimum of about 2.69% to a maximum of about 3.86% (Figure 1). Subsequently, the milk samples were divided into two groups as group High (3.34 ± 0.10%) and group Low (2.86 ± 0.05%) (*p* < 0.01).

### 3.2. Differentially Expressed Proteins in Isolated MECs

**Figure 2:**
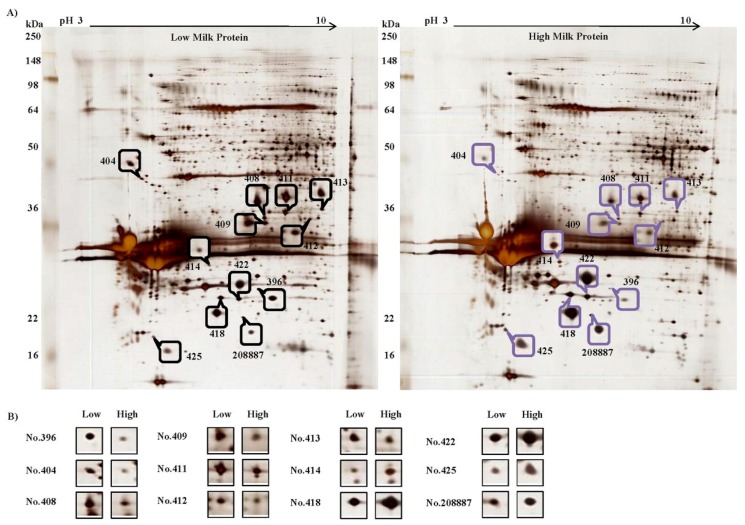
Representative two-dimensional gel electrophoresis (2-DE) images of isolated milk epithelial cells (MECs) (**A**) and differently expressed proteins (**B**).

In the isolated MECs, there were more than 450 detectable spots in each of the 2-DE images and ten differently expressed proteins were identified (Figure 2). Seven of them were down-regulated (heat shock protein beta-1 (HSPB1), 78 kDa glucose-regulated protein (GRP-78), l-lactate dehydrogenase B chain (LDH-B), malate dehydrogenase, cytoplasmic (MDH1), annexin I (ANXA1), cytokeratin-7 (CK-7), and glyceraldehyde-3-phosphate dehydrogenase (GAPDH)), whereas three were up-regulated in MECs of group High (prohibitin (PHB), beta casein (CSN2) (Spot 418 and 422), and alpha S1 casein (CSN1S1)) (Spot 425 and 208887)) (Table 2).

### 3.3. Effect of l-Leucine Depletion on Total Proteins Content in MAC-T Cells

**Figure 3:**
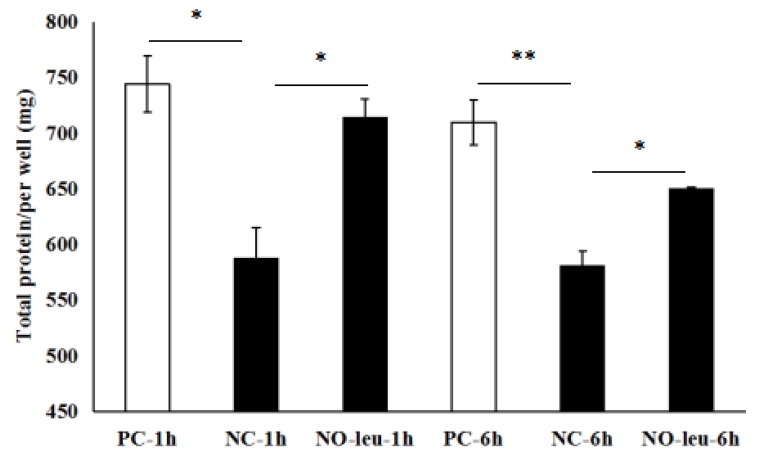
Effects of l-leucine limitations (1 h, 6 h) on total proteins content in mammary alveolar cells (MAC-T cells). NC: Negative control, Earle’s Balanced Salts Solution + insulin (5 μg/mL) + hydrocortisone (1 μg/mL) + prolactin (5 μg/mL); PC: Positive control, NC medium supplemented with 0.70 l-arginine, 0.15 l-histidine, 0.42 l-isoleucine, 0.45 l-leucine, 0.5 l-lysine, 0.12 l-methionine, 0.22 l-phenylalanine, 0.45 l-threonine, 0.04 l-tryptophan, and 0.45 l-valine (all in mmol/L); NO-leu: PC media devoid of l-leucine. * *p* < 0.05, ** *p* < 0.01.

After both 1 h and 6 h incubation, the total proteins synthesis in MAC-T cells of group NC were significantly lower than those of the groups of PC (*p* < 0.05; *p* < 0.01) and NO-leu (*p* < 0.05; *p* < 0.05) (Figure 3).

### 3.4. Differentially Expressed Proteins in Isolated MECs

**Figure 4:**
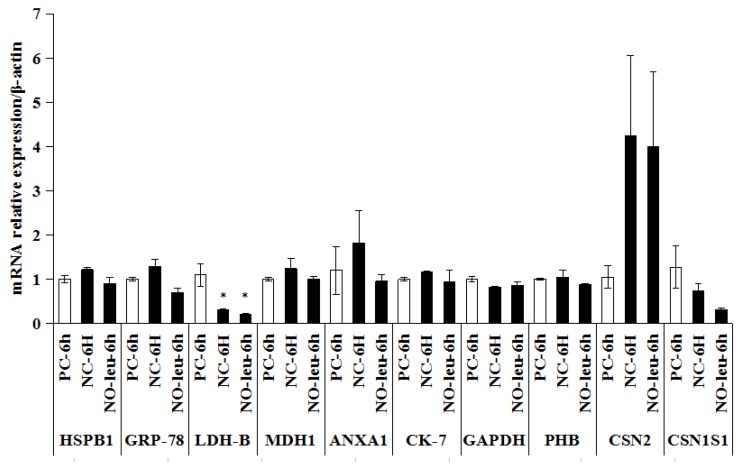
Verification test of candidate proteins obtained from isolated milk epithelial cells (MECs) in MAC-T cells after l-leucine limitations for 6 h by real time PCR. NC: Negative control, Earle’s Balanced Salts Solution + insulin (5 μg/mL) + hydrocortisone (1 μg/mL) + prolactin (5 μg/mL); PC: Positive control, NC medium supplemented with 0.70 l-arginine, 0.15 l-histidine, 0.42 l-isoleucine, 0.45 l-leucine, 0.5 l-lysine, 0.12 l-methionine, 0.22 l-phenylalanine, 0.45 l-threonine, 0.04 l-tryptophan, and 0.45 l-valine (all in mmol/L); NO-leu: PC media devoid of l-leucine. * *p* < 0.05. There were 3 biological samples in each group and the trial was performed in triplicate.

The mRNA expression of LDH-B was found to be significantly down-regulated in both groups of NC (*p* < 0.05) and NO-leu (*p* < 0.05). No significant difference was found regarding the other genes (Figure 4).

## 4. Discussion

The present data indicated significant differences existed in the milk protein synthesis ability of individual cows, even when fed the same diet. Similar to a previous report, this study showed that the milk protein levels of 25 multi-lactation Holstein cows (6–12 weeks lactation) were ranging from 2.55% to 3.30% [2]. The milk samples were divided into two groups according to their protein contents and ten differently expressed proteins were identified in the isolated MECs using a 2-DE system. HSPB1 was suggested to play a role in testosterone-related myogenesis in beef cattle [12], and a negative association of its expression with meat tenderness was observed in Nellore cattle [13]. ANXA1 was previously reported to be expressed in mammary glands of lactating cows [14] and have a negative connection with milk *cis*-9, *trans*-11 CLA level in dairy cows [11]. LDH-B, an enzyme involved in cellular carbohydrate metabolic process and glycolytic process, can catalyze reversible conversion of pyruvate to lactate as it can convert NADH to NAD^1+^ and back. In the present study, the protein expression of LDH-B was less in MECs from cows with high protein level than that from cows with low protein level. In addition, LDH-B is down-regulated in adipose tissue from Korean bulls relative to steers [12], and LDH-B was reported as a tenderness marker protein for goat muscle [15]. Both CSN2 and CSN1S1 are specific milk proteins secreted by mammary gland cells. CSN2 might be related to mammary protein synthesis [16], and allele CSN2 B had the effect of increasing β-casein content and decreasing content of αS1-casein [17]. A previous study has also indicated that genotype of CSN3, another milk protein, can significantly affect milk yield and composition in dairy cows [4]. However, whether CSN2 and CSN1S1 have similar functions remains unknown. For the other five proteins, little information was found on their roles involved in milk protein synthesis. GRP-78 is involved in protein folding and assembly [18]. MDH1 is an oxidoreductase that plays a part in tricarboxylic acid cycle [19]. CK-7 can stimulate DNA synthesis in cells [20]. GAPDH is an energy metabolism-related enzyme in the glycolytic pathway [21], whereas PHB has a role in regulating proliferation [22].

l-leucine is recognized as a major regulator of milk protein synthesis [8,16,23]. In this study, our data proved that l-leucine depletion could decrease milk protein content in MAC-T cells; in accordance with some previous reports showing that depletion of leucine could reduce protein synthesis [7,24]. In order to improve the understanding of leucine-specific signaling in milk protein synthesis, the association of these genes with l-leucine was checked in MAC-T cells but not in MECs due to limitations in sample availability. Interestingly, mRNA expression of LDH-B was significantly down-regulated when l-leucine was depleted. Moreover, it has been reported that l-leucine can inhibit oxidation of pyruvate [25]. These data suggest that there is a positive association between l-leucine and LDH-B. It is well known that l-leucine can regulate protein synthesis through mTOR pathway [16,24]. Whether LDH-B regulates milk protein synthesis via the l-leucine-mTOR pathway remains unclear and needs further study. In addition, no significant difference was found regarding the other genes, which may be due to the possible random errors in the experiment.

## 5. Conclusions

Milk protein content and global protein expression were found to be significantly different in animals fed the same diet. LDH-B is negatively related to the milk protein content of dairy cows and has a positive relationship with l-leucine. Our findings may provide some theoretical basis to study individual difference in milk protein synthesis ability of dairy cows.

## Figures and Tables

**Table 1:** Primer sequences for candidate genes used in this study.

Gene ^1^	Accession Number	Primers	
ACTB	NM_173979.3	forward	GCGTGGCTACAGCTTCACC
		reverse	TTGATGTCACGGACGATTTC
HSPB1	NM_001025569.1	forward	GCCGGAAACAAGTAAAGACC
		reverse	GGTGAGGATGTCCAGTGATG
GRP-78	NM_001075148.1	forward	CTTCTCGGAGACCCTGACTC
		reverse	CACTTTCTGGACAGGCTTCA
LDH-B	NM_174100.1	forward	GTGGAGTGGAGTGAATGTGG
		reverse	TGTCTGTTCCCATTTCTGGA
MDH1	NM_001034628.2	forward	CAACCATGCCAAAGTGAAAC
		reverse	GCCAGCTGTCATCTTTCAGA
ANXA1	NM_175784.3	forward	TTCTTTGCTGAGAAGCTCCA
		reverse	CAGAGCGGGAAACCATAATC
CK-7	NM_001046411.1	forward	CATGAACAAGGTGGAGTTGG
		reverse	AGCTCTTTCAGCTCCTGCTC
GAPDH	NM_001034034.2	forward	CGTTCGACAGATAGCCGTAA
		reverse	TCACCATCTTGTCTCAGGGA
PHB	NM_001034572.2	forward	GAGATCCTCAAGTCCGTGGT
		reverse	ACCAGCTCTCTCTGGGTGAT
CSN2	NM_181008.2	forward	GTGAGGAACAGCAGCAAACA
		reverse	TTTTGTGGGAGGCTGTTAGG
CSN1S1	NM_181029.2	forward	GCTGAGGAACGACTTCACAG
		reverse	AGGCCAGTTCCTGATTCACT

^1^ ACTB β-Actin, HSPB1 Heat shock protein beta-1, GRP-78 78 kDa glucose-regulated protein, LDH-B l-lactate dehydrogenase B chain, MDH1 Malate dehydrogenase cytoplasmic, ANXA1 Annexin I, CK-7 Cytokeratin-7, GAPDH Glyceraldehyde-3-phosphate dehydrogenase, PHB Prohibitin, CSN2beta casein, CSN1S1 alpha S1 casein.

**Table 2:** List of differently expressed proteins (≥2-fold or ≤0.5-fold) in isolated MECs.

Spot No.	UniProtKB/Swiss-Prot Entry	Protein Name ^1^	Theory; Calculation Mol. Mass (kDa)*/PI*	MASCOT Score	Peptides Matched	Sequence Coverage (%)	Molecular Functions	Protein Expression (Area)		
								H	L	H/L
396	Q3T149	HSPB1	22.4/5.98;	750.45	233	60.70	Stress resistance and actin organization	0.0423	0.2046	0.207
			22.4/6.40							
404	Q0VCX2	GRP-78	72.4/5.07;	131.03	38	10.53	Facilitating the assembly of multimeric protein complexes inside the ER	0.0242	0.2217	0.109
			72.4/5.16							
408	Q5E9B1	LDH-B	36.7/6.02;	150.54	56	17.37	Oxidoreductase in cellular carbohydrate metabolic process and glycolysis	0.0934	0.3852	0.242
			36.7/6.44							
409	Q3T145	MDH1	36.4/6.16;	20.44	9	3.59	Oxidoreductase in cellular carbohydrate metabolic process and malate metabolic process	0.1291	0.5164	0.250
			36.4/6.58							
411	P46193	ANXA1	39.0/6.37;	2103.24	522	46.24	Plays important roles in the innate immune response and has anti-inflammatory activity	0.2705	0.6448	0.420
			39.0/6.81							
412	Q29S21	CK-7	51.5/5.79;	59.14	17	2.58	Blocks interferon-dependent interphase and stimulates DNA synthesis in cells	0.0742	0.1984	0.374
			51.5/5.97							
413	P10096	GAPDH	35.8/8.51;	17.58	7	7.93	Oxidoreductase and transferase in apoptosis glycolysis, translation regulation	0.1343	0.3234	0.415
			24.2/8.53							
414	Q3T165	PHB	29.8/5.57;	703.84	225	52.57	Has a role in regulating proliferation	0.1909	0.0777	2.457
			29.8/5.76							
418	P02666	CSN2	25.1/5.26;	224.15	61	19.14	Antioxidant activity; negative regulation of catalytic activity; transporter activity	0.8430	0.3449	2.444
			23.6/5.34							
422	P02666	CSN2	25.1/5.26;	106.40	30	19.14		0.6392	0.2882	2.218
			23.6/5.34							
425	P02662	CSN1S1	24.5/4.98;	94.57	41	19.83	Antioxidant activity; transporter activity; important role in the capacity of milk to transport calcium phosphate	0.2055	0.1024	2.008
			13.8/5.55							
208887	P02662	CSN1S1	24.5/4.98;	79.25	32	19.83		0.2066	0.0820	2.518
			13.8/5.55							

^1^ Heat shock protein beta-1, GRP-78 78 kDa glucose-regulated protein, LDH-B l-lactate dehydrogenase B chain, MDH1 Malate dehydrogenase cytoplasmic, ANXA1 Annexin I, CK-7 Cytokeratin-7, GAPDH Glyceraldehyde-3-phosphate dehydrogenase, PHB Prohibitin, CSN2 beta casein, CSN1S1 alpha S1 casein.
